# Emotional Intelligence and Personality Traits Based on Academic Performance

**DOI:** 10.3389/fpsyg.2022.894570

**Published:** 2022-06-14

**Authors:** Xin Dong, Olga A. Kalugina, Dinara G. Vasbieva, Arslan Rafi

**Affiliations:** ^1^School of Marxism, Zhuhai College of Science and Technology, Zhuhai, China; ^2^Department of English for Professional Communication, Financial University Under the Government of the Russian Federation, Moscow, Russia; ^3^Financial University Under the Government of the Russian Federation, Moscow, Russia; ^4^Department of Leadership and Management Studies, Faculty of Contemporary Studies, National Defence University, Islamabad, Pakistan

**Keywords:** virtual experience, emotional intelligence, personality traits, academic performance, students performance

## Abstract

The purpose of this study was to examine the role of personality traits on academic performance. Furthermore, this study also aims at exploring the effects of virtual experience (mediator) and emotional intelligence (moderator) between personality traits and academic performance of the students. The findings imply that personality traits are the strong predictors of better academic performance. However, several personality traits do not have a positive impact on the academic performance. The study further suggests that students who have emotional abilities and virtual experience are more likely to perform well in their academics. The population of this research consists of students in various colleges and universities in developing regions. Thus, the sample consists of bachelor's and master's students. Existing scales are adopted with minor changes to make it more suitable and understandable within the study context. A total of 319 questionnaires were distributed. Among these 365 questionnaires, 234 questionnaires were received and further used for the purpose of data analysis. This shows an encouraging response from the targeted sample. Education and productivity of the students are influenced by their personality as well as their emotional intelligence abilities. The findings imply that being extrovert is a strong predictor of student achievement and should be prioritized in intervention strategies. This personality feature is responsible for performance in addition to virtual learning experience. Despite its low overall relative value, agreeableness is a significant driver of student achievement. Along with ability and aptitude assessments, personality evaluations might be utilized as a secondary screening tool to identify adolescents at risk of underperformance and academic performance failure. Therefore, learning emotional skills would be beneficial to cope the modern challenges of the competitive educational environment. Virtual experience and being emotionally sound can help students to learn quickly and to be more adaptive into the new world of digitalization. The conclusions of the current study have significant consequences for educators and policymakers. They must accept that boosting emotional intelligence levels through teaching or training is a significant objective of contemporary education. The emotional intelligence abilities of the students related to culture may be shown in a variety of ways, from expectations toward students to interpersonal interactions with students, and from teaching techniques to evaluation methods.

## Introduction

It has been observed that virtual reality, which is the most accurate representation of manufactured reality, is becoming increasingly popular. In more detail, virtual reality depicts events or things that are knotted to computer technology in order to create the illusion of an interactive, three-dimensional world in which objects that have the appearance of spatial presence can be seen. Critically speaking, virtual experience predominantly has different aspects to interpret the virtual experience eventually (Burdea and Coiffet, 2003). On the contrary, virtual reality has a great influence on emotional intelligence (EI). The ability to sense, control, and assess emotions is referred to as EI. Some academics believe that EI can be taught and improved, while others believe it is a natural trait.

Furthermore, EI is built on the foundation of three complimentary competency modules, namely, psychological, social, and pragmatic. There is also a grave and unavoidable relation of virtual experience and EI. Interestingly, virtual experience and EI have a core relation with personality traits principally. Talking about personality traits, personality is the set of thoughts (Back and Kandler, 2020), attitudes, and emotional attributes (Markiewicz et al., 2020), which bonds to social actions that create influential environment. Personality is a significant determinant of life outcomes (Almlund et al., 2011) and traits of personality can be evaluated while probing the academic performance eventually. It has also been observed that the role of personality traits is very much sway to the academic performance (Mammadov, 2022). Academic performance is regarded as crucial for job pathways, individual life trajectories, and long-term success. It is also regarded as important as a societal effect. Personality is one of the non-cognitive characteristics that have been systematically linked to academic achievement.

Moreover, there is a significant relation of personality traits that have an important impact on academic performance, and is currently being vital. Continuing the theme, virtual experiences have a major impact on academic performance as well. Furthermore, EI is likely to mediate the interplay between students' personality traits and virtual experiences that convey persuasiveness and motivation. More research is needed to determine how conscientiousness, a personality attribute linked to higher academic success, is linked to academic performance.

It is a pragmatic fact that academic performance is well influenced by the personality traits eventually (Gatzka and Hell, 2018). Moreover, it could be more appropriate to conceive of strengthening productive personality as a more beneficial intervention during the early years of education. More objectives of the personality traits can also have more impacts on the academic performance of the students. Universal five big personality traits, such as consciousness, openness, agreeable, neuroticism, and extraversion, has an impression on the academic performance in different approaches.

Some personality traits, such as extraversion, agreeableness, and openness to new experiences emerged as important predictors of EI. Conscientiousness and neuroticism had no effect on EI eventually. Ultimately, EI has a great impact on academic performance (Kokkinos and Vlavianou, 2021) that is evaluated as a moderate role in academia. Based on the academic performance, EI is thought to contribute to the advancement of thinking and the ability to control emotions in stressful situations. However, according to the trait EI theory, the construct should not have a significant and direct association with cognitive capacity or its close proxies, such as academic accomplishment (Petrides and Mavroveli, 2018).

Personality traits and academic performance are directly related (Mavroveli et al., 2009; Clark and Schroth, 2010). A previous study by Webb (1915), who minutely scrutinized academic performance, is considered to be vital. Moreover, HEXACO is a vital tool through which traits of personality can be ascribed well and it would be really convincing while correlating it to virtual experience and academic performance.

The prognostic influence of personality on preferences for academic attainment concludes the approaches of the high impact. Personality and preferences may act as indicators of future performance, so policymakers can assist children with negative personality traits or preferences. According to Fariba (2013), there is a link between personality qualities and learning styles, which could contribute to higher levels of learning and, as a result, a greater sense of self-satisfaction and enjoyment of the learning process. Predominantly, some learning outcomes are deliberated concerning to learning styles that highlight the core impacts of personality traits while connecting to academic performance.

This manuscript surrounds the academic performance and personality traits that have much of an influence based on academic performance. In this sense of understanding, five factor model (FFM) of personality trait is focusing at all. All the five major personality traits have a direct impact on academic achievements. Two among all the major personality traits, Conscientiousness and Emotional Stability, are vital ones, and rest of the three are merely discussed based on their reflection to the academic performance. It is known that cognitive ability always has a direct influence on whether academic performance is high or moderating. Moreover, individual tasks are well recognized by the cognitive ability and EI, which concerns the tasks within the team. These considerations can be best tools while doing research on personality traits and academic performance.

Ultimately, student's cognitive capacity may indicate what he or she can do, whereas a student's personality traits may replicate what they will do. Except cognitive capacity, cognitive ability, the relation of both conscientiousness and emotional stability, means a lot while approaching to academic achievements. The FFM is the most prevalent model of personality structure in contemporary work examining personality traits and academic achievement. Extraversion, Neuroticism, Openness to Experience, Agreeableness, and Conscientiousness are assumed to encompass all of the more limited personality traits that occur at lower levels of the personality hierarchy in this paradigm. More focusing on conscientiousness, this research focuses on the academic performance, as conscientiousness is directly related to achievements.

Furthermore, McElroy et al. (2007) also test UTAUT and TAM to highlight personality traits and academic performance based on relationship of technology with the mentioned variables. It is clearly denoted that personality traits have a relation with technology and the UTAUT model already confirmed it. There is different responsiveness of the personality traits with the accordance of different conditions. Professionals with a high conscientiousness score are goal-oriented and more inclined to accept and apply new technology. Moreover, the conscientiousness, among all other personality traits, shows a significant relationship with academic performance and its impact is high compared with other traits.

According to our analysis of the research, if the content is rich and applications of the technology are upright as well as up to the mark regarding students' inclination toward learning, technology plays a vital role concerning personality traits and academic performance (Jacques et al., 2009). There is much competition of the technology and its application if academic performance is targeted to enhance in academia. There is also a trendy approach of the technology as an intervention that blended math and science educational videos with infrastructure changes, which are the best tools to improve the educational performance. Furthermore, technology can help learners in rural locations have access to education. The evidence for these programs is promising. Even remote education is handy while relating technology to academic performance.

## Research Questions

When it comes to virtual experience and its relationship with EI, how and by what means personality traits have a substantial impact on academic performance is still being sought out. Continuing with the discussion, virtual experiences have a significant impact on academic performance. Such a phenomenon, which indicates another critical consideration, such as the fact that both emotional intelligence and academic performance are observed to be in a predominantly positive relationship, is particularly vulnerable to discussion. Furthermore, EI is expected to have a role in moderating the interaction between personality traits and virtual experiences of the students that portray persuasiveness and motivational characteristics of the students.

### Novelty of the Research

In this research study, the best ground is that we strongly focus EI and its relationship with virtual reality based on academic performance. In this light, three sub-dimensions of EI, namely, managing relationships, integrity, and self-development, have a high positive link with academic performance, and ultimately, virtual reality is related to this phenomenon. EI has been associated with more pro-social behavior, improved academic performance, and more empathy for students in this study.

In academia, EI has been significantly observed, and it has an excellent relationship with academic performance. Furthermore, the value of virtual experience in mediating intelligence quotient or overall mental capacity is well understood when it comes to professional performance. Along with virtual experience and emotional intelligence, academic performance, which is commonly regarded as a critical component of academic success for pupils, is also highly regarded. A lot of elements are still important to be highlighted considering the essence to EI and academic achievement. Similarly, it is vital to carve-out virtual experience while correlating it into the dual phenomenon of EI and academic performance predominantly; it has also been assumed that conscientiousness, one of the personality traits, is the best predictor of academic achievement across the board, accounting for five times as much variance in grades as the intelligence quotient. Overall academic success is predicted by agreeableness, conscientiousness, and openness while glancing into the important facts about virtual experience ultimately.

Moreover, different models and theories are also candid aspects of this research. The Honesty-Humanity, Extraversion, Agreeableness, Conscientiousness, and Openness to Experiences (HEXACO) is a vital tool through which traits of personality can be ascribed well and it would be really convincing while correlating it into virtual experience and academic performance. Importantly, academic performance theory, model of EI, and moderation role of EI are innovative features of this research and are also valuable. With the conduction of this research, strong relationship of the personality traits, academic performance, and virtual experience are important aspects to be considered ventures.

### Literature Review

It has been observed that talking about different thinking pattern, actions, and feelings, personality traits count well into it (Blickle, 1996; Soutter et al., 2020). Regarding personality traits and definite structure, then, the conversational Big Five personality traits are a perfect source to be considered (Roccas et al., 2002; O'Connor and Paunonen, 2007). There is wholesome of the personality traits and essence of the academic performance while observing both with the help of stipulated models. Openness is defined as a person's proclivity for intellectual curiosity, active imagination, and responsiveness to feelings, and aesthetic sensibility (Saklofske et al., 2012). The disposition to be orderly, ambitious, determined, dependable, and purposeful is known as conscientiousness.

Major et al. (2006) observed that the three personality traits, such as conscientiousness, openness, and extraversion, are best that consolidate as being predicted motivation for learning and, contrary to it, there is an unappealing as well as an uninviting relation of the neuroticism based on learning attitude. Neuroticism is linked to a person's proclivity for unpleasant emotions such as guilt, rage, fear, disgust, grief, and shame. The propensity to be chatty and aggressive, extraversion is another trait with different essence of learning phenomenon. Talking about consensus on this approach in terms of cooperativeness and altruistic nature, it has different conscientiousness with the context of learning attitude based on academic performance. One of the most widely accepted personality models is the Five Factor Model (Barrick et al., 2003). One of the most significant benefits of employing the Big Five model is that personality does not change significantly and remains steady across age groups over a 4-year period. There have been numerous studies on the relationship between academic success and personality.

According to Hodson et al. (2009), the model is unique and has been considered by researchers while connecting it to personality traits. Furthermore, multiple studies have found that the Big Five personality traits have a considerable impact on student academic achievement because of the many methodologies used to measure academic success around the world. Similarly, there are regional disparities in the intervals used to measure academic performance and personality traits. Students pursuing different degree programs or from different nations, locations, or situations may have distinct aptitudes, personalities, and learning behaviors.

According to Simpraga et al. (2021), personality traits are the patterns of thoughts, feelings, and behaviors that people have. The basic dimensions of traits of the persons are highlighted in the trait theory. In this sense of understanding, consistency, individual differences, and stability are the vital judgmental tools as well. Sigmund Freud, a famous psychiatrist, established the psychoanalytic personality theory. A person's personality, according to Freud, is the sum of their intrinsic inclinations and familial influences. Katherine Cook Briggs and Isabel Briggs Myers, a mother and daughter, created the humanistic personality theory. The necessity of self-growth in developing healthy personality qualities is highlighted by humanistic personality theory. The exam was created by the researchers in order to better understand personality characteristics.

Hughes and Smith (1990) believe that academic performance is the assessment of a student's ability in a variety of academic areas. Personality qualities were found to be highly connected to academic achievement. Furthermore, conscientiousness was the most important predictive variable, accounting for the variance in academic attainment. Moreover, Elger (2007) developed the theory of academic performance. Six core concepts are emphasized in the theory to establish a framework that may be utilized to explain performance as well as performance improvements. Producing valuable results is what it means to perform.

H1: *Personality traits has a positive significant impact on academic performance of the students*.

“Virtual reality is the computer-generated version of real life. Computer programmes deliver a visual environment through a TV headset that may pixel-perfectly match the actual world—or show a wholly fictional one”. Virtual reality differs from augmented reality, which combines computer-generated information such as photos, text, movies, animation, and music with a real-world, real-time image (viewed through a cellphone camera; Lund and Agbaji, 2018).

Many areas, including medical research, engineering, architecture, product development, and geology, have utilized virtual reality to visualize and analyze abstract ideas (Portman et al., 2015; Alhalabi, 2016; Tudor and Minocha, 2018). According to this study, employing virtual reality as a teaching tool enhances students' knowledge of ideas and test results dramatically.

Libraries focus on integrating learning-friendly technology to everyday instructional activities, enhancing access to research help, and expanding available information resources to users as part of their essential mission of generating chances for learning and promoting education in society (MacWhinnie, 2003). The feasibility has been investigated by a number of researchers. In libraries, virtual reality is being used. Poulter (1993) introduced the idea of an online catalog—a virtual reality library—that would allow users to search for books online. Within a computer-generated environment, users may explore an information area and order things from shelves. This online catalog gave consumers access to information resources that either did not have a physical repository or did not have one that was physically accessible to them (such as in off-site storage). After Second Life, an online virtual world that allows people to interact with one another, a virtual library project—the Virtual Library Project—was established in 2003, and it allows educational institutions to perform teaching and research activities. Alliance Library System and OPAL launched Second Life Library 2.0 (Swanson, 2007). It offered a variety of library services, including synchronous and asynchronous access to library collections and databases, as well as to real-world individuals *via* their virtual avatars Communication that is not synchronous. The users of this service expressed a high level of satisfaction.

H2: *Virtual experience is likely to mediate the relationship between personality traits and academic performance*.

Users might utilize the catalog to access information resources that either did not have a physical repository or did not have one that was up to date. Controlling one's emotions under atypical settings is a key component of emotional intelligence. Emotional intelligence is now commonly recognized as a significant barrier to professional success and the growth of one's personality. Urquijo and Extremera (2019) explain that when emotions are added up, you get personality traits ultimately. Emotional intelligence has recently been studied by another psychologist, Goleman and Gurin (1993). Emotional intelligence can now be measured and correlated with a person's performance, thanks to new research. It is a set of skills, attitudes, talents, and competencies that determine the individual's behavior, reactions, state of mind, coping style, and communication style.

Moreover, knowing persons' quality and psychological outcomes, some theories are best sources to study on. Acting, feeling, and essence of working are the prominent aspects of the persons' personality (Weiner, 2005). With the help of different theories related to personality traits, different modes of the emotions and personality positions can also be judged. Generally, personality's angles are stable, but some other angles of outer variables can amend the personality traits. Different personality can also be judged while trying to determine all aspects of the personality. In this sense of understanding, there are some important personality theories that highlight the personality trait well, such as trait perspective, psychoanalysis, humanistic, trait perspective, and behaviorist theory. Through these personality theories, the personality development, different aspects of personality, and stimulation can be denoted perfectly.

On the contrary, EI is also vital, as it has a direct relation with personality traits and academic performance, as emotional determination is accurate personality judgment that denoted best tools (Chrusciel, 2006). For driving inner thoughts in others' personalities, the people with high EI can also observe other emotional consideration. The definition of EI proposed by Mayer et al. (2008) tries to fit EI into the traditional criteria for a new intelligence. The model by Daniel Goleman emphasizes EI as a broad set of talents and skills that drive leadership effectiveness.

H3: *Emotional intelligence is likely to influence academic performance*.

The five primary EI structures are outlined in Goleman's model. Goleman also highlights all kinds of abilities of the persons' emotions and some of the emotional skills can also let the persons approach toward extraordinary outcomes. Individuals are born with a general EI, according to Goleman, which determines their ability to gain emotional competences. Self-awareness, self-regulation, motivation, empathy, and social skills are the five components of Daniel Goleman's EI hypothesis (Goleman, 2001, 2014). EI can be used to achieve objectives and build a happier and healthier workplace atmosphere.

Our manuscript prolongs the personality traits and its impact on academic performance. In this sense of understanding, personality traits and their connection with academic performance is the focus of this research as well. Accompanied by personality traits, EI and useful models can also predict this unique research. Personality traits' model, theories, academic theories, models of EI are also added to furnish this research predominantly.

H4: *Emotional intelligence is likely to moderate the relationship between personality traits and academic performance*.

## Methodology

The population of this research consists of college and university students studying in developing regions. Thus, the sample consists of bachelor's and master's students. Existing scales are adopted with minor changes to make it more suitable and understandable within the study context. For instance, the scale for academic performance is selected from George and Rapport. For measuring emotional intelligence, 15-items scale of Kidwell et al. (2011) is adapted. For personality traits, NEO-PI 15-items scale is used. Finally, virtual experience scale is adapted from Chertoff et al. (2010). Several questions regarding demographics were included in the questionnaire. Hence, this research study used non-probability sampling in which further convenient sampling is selected. However, before using this questionnaire, Reliability and Validity were established in the local context. A Google-form based questionnaire was designed, and the link was shared with the students. Moreover, data have also been obtained with the consent of multiple colleges/universities in developing regions. The authenticity of the data was confirmed by the participants who were involved in the student community. There were many complexities faced during collection of the data due to the outbreak of COVID-19. However, the consistent follow-ups have helped the researchers to collect 234 filled questionnaires from the students studying in different colleges and universities of developing regions. The first two authors, who are currently residing in developing regions, suggested that fellow academic students from developing regions colleges/universities participate in the survey and share the link with other users on their regular community meet-ups. Because of these frequent meet-ups and the backing of the academic community, the current study was able to achieve a high response rate. Demographics and Normality of the data is checked with the help of Statistical Software of Social Sciences (SPSS) version 21. Once the data normality is confirmed, composite reliability, Convergent validity, Discriminant validity, and hypotheses testing is done with the help of Structural Equation Modeling. For mediation analysis, mediation assumptions by Baron and Kenny are followed.

## Results and Analysis

A total of 365 questionnaires were distributed. Among these 365 questionnaires, 234 questionnaires were received and further used for the purpose of data analysis. This shows an encouraging response from the targeted sample. Most of the respondents come in the age range of 25–35 years, which uses these social media platforms once in 6 months. Reliability and normality of data is examined with the help of SPSS version 21.

### Scale Validity and Reliability

The construct reliability and convergent validity are examined with the help of statistical software AMOS version 20. Big Five personality traits is examined on a 15-item scale and the factor loadings were quite good except second items of Neuroticism dimensions. Composite reliability and average variance extracted values for each personality trait are well above the minimum criteria. For academic performance, the convergent validity is also above the minimum criteria; the value for average variance extracted is 51%, which is quite above the minimum criteria of 50%. Emotional intelligence concept is measured on newly developed 15-item scale; however, the items which have low factor loading scores are excluded from the final analysis. Finally, for virtual experience, the concept is measured on a 6-item scale, and the factor loading scores, reliability, and validity are quite good. The value for composite reliability is 0.854, and average variance extracted is 0.595 as shown in Table 1.

**Table 1:** Factor loadings.

**Variable**	**Item code**	**Loadings**	**Composite reliability**	**AVE**
Neuroticism	NEU1	0.807	0.879	0.785
	NEU3	0.959		
Extrovert	EXT1	0.870	0.907	0.765
	EXT2	0.820		
	EXT3	0.931		
Openness	OPEN1	0.903	0.884	0.718
	OPEN2	0.900		
	OPEN3	0.728		
	AGREE1	0.762	0.833	0.624
Agreeableness	AGREE2	0.770		
	AGREE3	0.836		
	CON1	0.850	0.827	0.622
Conscientiousness	CON2	0.901		
	CON3	0.576		
	COP1	0.762	0.862	0.610
	COP2	0.854		
	COP3	0.733		
	COP4	0.770		
	AP1	0.514	0.721	0.512
Academic performance	AP2	0.642		
	AP3	0.615		
Emotional intelligence	EM6	0.641	0.893	0.513
	EM7	0.766		
	EM8	0.752		
	EM9	0.710		
	EM10	0.692		
	EM11	0.743		
	EM12	0.796		
	EM13	0.609		
Virtual experience	VP1	0.709	0.854	0.595
	VP2	0.779		
	VP3	0.807		
	VP4	0.767		

The discriminant validity is examined with the help of square root of AVE, and it must be greater than the correlation values between the latent variables. For instance, the discriminant value for virtual experience is 0.771 and it is above the correlation values between latent variables. Similarly, personality traits discriminant validity value is 0.936 and Emotional intelligence discriminant value is 0.715; these values are well above the correlation values between latent variables as shown in Table 2. Hence, the convergent and discriminant validities are statistically significant for this scale.

**Table 2:** Discriminant validity.

**Variable**	**(1)**	**(2)**	**(3)**	**(4)**
Virtual experience (1)	0.771			
Personality traits (2)	0.362	0.936		
Academic performance (3)	0.664	0.779	0.715	
Emotional intelligence (4)	0.179	0.218	0.246	0.716

*Diagonal elements (bold figures) are the square root of the AVE (the variance shared between the constructs and their measures). Below-diagonal elements are the correlations among variables*.

### Structural Model Analysis

Regression analysis explains the impact of independent variable on the dependent variable. In this particular research study, Big Five personality traits (SMM) is taken as an independent variable, whereas academic performance is taken as a dependent variable. First hypothesis is about the impact of personality traits on academic performance. Result revealed that personality traits have a positive significant impact of 79%, whereas the (Sig. < 0.05) impact on student's academic performance. The results also showed a positive significant relationship between virtual experience and academic performance. In simple words, if the virtual experience is enhanced by 1%, it would have a positive significant impact of 66%, whereas the (Sig. < 0.05) positive impact on academic performance of the students. In the third hypothesis, mediation impact of virtual experience between personality traits and academic performance is examined. Results confirmed partial mediation effect of virtual experience between personality traits and academic performance. In fourth hypothesis, direct impact of emotional intelligence on academic performance of the student is examined. Results revealed that emotional intelligence has a positive significant impact of 47%, whereas the (Sig. < 0.05) on academic performance of the students. Finally, the moderating role of emotional intelligence is examined between personality traits and academic performance. Emotional Intelligence contingent impact enhanced the relationship between personality traits and academic performance of the students.

## Discussion and Conclusion

Results revealed that emotional intelligence has a positive significant impact of 47%, whereas the (Sig. < 0.05) on academic performance of the students as shown in Table 3. However, extroverted people are high-energy and talkative, and they like it. Extroverts are continuously seeking to meet new people; they do not hesitate to introduce themselves to strangers, and their energy level is quite high. They rarely avoid uncomfortable circumstances for the fear of messing up or encountering academic pressure. Highly extroverted people enjoy socializing with others; they are comfortable to expose their self in a group situation and frequently experience positive emotions such as excitement and enthusiasm. Similarly, this personality trait describes people who are cooperative, sociable, and sympathetic. Warmth, friendliness, and tact are typical characteristics of someone with a high level of agreeableness. They often have a positive outlook on other people and are eager to collaborate with them. They are willing to set their own interests aside for the sake of others. Low agreeableness makes people uncooperative, unfriendly, and distant. They always prioritize their own interests over the interests of others. Individuals who are disagreeable have a lesser concern for others and for the social rules of politeness.

**Table 3:** Hypothesis testing.

**Hypothesis**	**B**	**T**	***p*-value**	**Statistical decision**
H1: PT → AP	0.785	19.301	0.000	Supported
H2: VE → AP	0.664	13.513	0.000	Supported
H3: PT → VE → AP	0.715	18.667	0.000	Supported
H4: EI → AP	0.467	8.051	0.000	Supported
H5: PT*EI → AP	0.712	15.425	0.000	Supported

*PT, personality traits; AP, academic performance; VE, virtual experience; EI, emotional intelligence*.

Furthermore, people who are conscientious are dependable and punctual. It symbolizes productivity and accountability; highly conscientious people desire order and organization in their work in order to attain their goals. People with high conscientiousness are eager and devoted to completing their duties, whereas those with low conscientiousness are calm, relaxed, and show little motivation to finish their duties and tasks.

On the one hand, while introvert are those people who always try to escape from offices and their homes, they are quiet, calm, and low extroversion. They keep themselves away from social gathering. On the other hand, findings also suggest that people who are very creative and continually striving to open new doors have a strong desire to take on new tasks and learn new things. People who have a high level of this attribute appreciate trying new things and going on adventures. Therefore, the findings suggest that students with this personality traits are likely to have a slight positive impact on their academic performance.

This study compared the perceptions of a virtual experience procedure to “live” class formats in terms of learning level, sense of coherence, and social and task interaction. Since the “comparison with live course” was done on a five-point Likert scale, divergence of replies from the mean in questions has a middle value of “roughly the same.” It is possible to measure a position that is “roughly the same.” This clearly reveals that participants' assessments of feeling of coherence, social contact, and task interaction are much lower than those of a “live” class. However, assessments of capacity to acquire new information differ only little from those in the “live” class. Simply expressed, there is a noticeable reduction of social processes in virtual experience designs; nevertheless, this degradation did not have a major impact on perceived learning capacity.

It was expected that emotional intelligence and academic success of students would have a statistically significant beneficial association. Higher levels of emotional intelligence, agreeing to previous research, should predict higher academic grades (*via* the capacity to cope with stresses such as assessment and evaluations, group dynamics, and the social and emotional demands of academic life). These data support prior research contention that EI is a distinct, quantifiable kind of ability. This intelligence may always correspond well with academic intelligence. Furthermore, the findings of this research study suggested that a high level of emotional intelligence can also improve learning from virtual experiences, which can positively affect the overall academic performance of the students.

## Conclusion

Education and productivity of the students are influenced by their personality as well as their emotional intelligence abilities. The findings imply that being extrovert is a strong predictor of student achievement and should be prioritized in intervention strategies. This personality feature is responsible for performance in addition to virtual learning experience. Despite its low overall relative value, agreeableness is a significant driver of student achievement. Along with the ability and aptitude assessments, personality evaluations might be utilized as a secondary screening tool to identify adolescents at risk of underperformance and academic performance failure. Therefore, learning emotional skills would be beneficial to cope with the modern challenges of the competitive educational environment. Virtual experience and being emotionally sound can help students to learn quickly and to be more adaptive into the new world of digitalization.

### Practical Implications

The conclusions of the current study have significant consequences for educators and policymakers. They must accept that boosting emotional intelligence levels through teaching or training is a significant objective of contemporary education. Emotional intelligence abilities of the students related to culture may be shown in a variety of ways, from expectations toward students to interpersonal interactions with students, and from teaching techniques to evaluation methods. Furthermore, personality traits have a huge influence on the academic performance. As this research study suggested that several personality traits have a positive influence on academic performance, *vis-a-vis*, there are some personality traits that might have a negative impact on the academic performance of the students. However, proper counseling and guidance can help to cope with personality-related issues, and hence can improve their overall personalities. This illustrates that the creation of virtual experiences may have a large and favorable impact on students' academic achievement. To the university classroom, bring your personality traits and experiences.

### Significance of the Study

It is significant to authenticate the HEXACO personality traits model. Furthermore, because of the many methodologies used to measure academic success around the world, the association of the EI and academic performance is unanimous undoubtedly. Students pursuing different degree programs or from different nations, locations, or situations may have distinct aptitudes, personalities, and learning behaviors. This study is an important resource for determining the relationship between virtual experience and EI based on academic performance. Furthermore, depending on academic success, EI and cognitive ability are important. Students' academic achievement or performance has a strong link to their personality attributes.

Furthermore, the majority of research suggests that personality traits have a significant impact on the academic performance. Specifically, conscientiousness is linked to consistent connection toward academic achievement/performance, which is really essential. Based on virtual experience and its relationship with personality traits improvement as well as enhancement in academic performance, it is really a great venture if involving the virtual experience into the said aspects of this research. This research is exclusive, as the core of the personality traits, specifically conscientiousness, agreeableness, and openness with their impacts on academic performance are the focusing aspects through the consideration of virtual experience.

The most powerful predictor, conscientiousness, which is one of the important personality traits that strongly connects to the academic achievements, is also concentrated. The findings of this research would definitely provide a key regarding how conscientiousness is related to academic achievements and sustained in its essence while considering academic performance. Here, personality traits model HEXACO is a key to venture it.

### Limitations and Future Research Directions

Although the results of this current study are very promising, but it has few limitations, for instance, first, this study consists of cross-sectional data collected during the period of COVID-19. Therefore, it is suggested to reconduct the same study once the COVID-19 pandemic is over. Second, reporting own emotional abilities maybe biased; therefore, Goleman emotional intelligence model can also be tested in students' academic performance context. When examining student academic performance, researchers can no longer ignore the problem of competition. A recent study has found links between culture, competition, and performance, as well as the relationship between competitiveness and performance. Additional research examines conscientiousness, a personality trait that has been shown to be associated with superior academic success, and specifically how conscientiousness has been shown to be associated with academic performance eventually. Another study identified significant cultural variations and demonstrated the importance of competitive mindset in driving performance. Therefore, future research must be conducted with these directions in another region to have a better understanding of this theoretical model.

## Data Availability Statement

The raw data supporting the conclusions of this article will be made available by the authors, without undue reservation.

## Ethics Statement

Ethical review and approval was not required for the study on human participants in accordance with the local legislation and institutional requirements. Written informed consent for participation was not required for this study in accordance with the national legislation and the institutional requirements.

## Author Contributions

All authors listed have made a substantial, direct, and intellectual contribution to the work and approved it for publication.

## Funding

This study was supported by 2021 Guangdong Provincial Educational Science Planning Project (Higher Education Special) - Three-Micro Integration Research on the Life-based Teaching of Ideological and Political Courses in Colleges and Universities (No. 2021GXJK170) and Zhuhai University of Science and Technology 2020 Innovation Ability Cultivation Project Key Cultivation Project (Humanities and Social Sciences Class) - Research on the Innovation of College Students' Ideological and Political Education Model in the ‘Post-epidemic' Era (No. 2020XJCQ014). The publication is prepared within implementation of the state assignment 2021 for the Financial University under the Government of the Russian Federation.

## Conflict of Interest

The authors declare that the research was conducted in the absence of any commercial or financial relationships that could be construed as a potential conflict of interest.

## Publisher's Note

All claims expressed in this article are solely those of the authors and do not necessarily represent those of their affiliated organizations, or those of the publisher, the editors and the reviewers. Any product that may be evaluated in this article, or claim that may be made by its manufacturer, is not guaranteed or endorsed by the publisher.
